# Isolation and Molecular Identification of Virulence, Antimicrobial and Heavy Metal Resistance Genes in Livestock-Associated Methicillin-Resistant *Staphylococcus aureus*

**DOI:** 10.3390/pathogens8020079

**Published:** 2019-06-14

**Authors:** Chumisa C. Dweba, Oliver T. Zishiri, Mohamed E. El Zowalaty

**Affiliations:** 1 Discipline of Genetics, School of Life Sciences, College of Agriculture Engineering and Sciences, University of KwaZulu-Natal, Durban 4000, South Africa; ccdweba@gmail.com (C.C.D.); Zishiri@ukzn.ac.za (O.T.Z.); 2 Infectious Diseases and Anti-Infective Therapy Research Group, Sharjah Medical Research Institute and College of Pharmacy, University of Sharjah, Sharjah 27272, UAE; 3 Department of Infectious Diseases, St. Jude Children’s Research Hospital, 262 Danny Thomas Pl, Memphis, TN 38105, USA

**Keywords:** *Staphylococcus aureus*, methicillin-resistant, *mecA*, *mecC*, antibiotics, heavy metals, livestock, resistance, virulence, zoonotic one-health, epidemiology

## Abstract

*Staphylococcus aureus* is one of the most important pathogens of humans and animals. Livestock production contributes a significant proportion to the South African Gross Domestic Product. Consequently, the aim of this study was to determine for the first time the prevalence, virulence, antibiotic and heavy metal resistance in livestock-associated *S. aureus* isolated from South African livestock production systems. Microbial phenotypic methods were used to detect the presence of antibiotic and heavy metal resistance. Furthermore, molecular DNA based methods were used to genetically determine virulence as well as antibiotic and heavy metal resistance determinants. Polymerase chain reaction (PCR) confirmed 217 out of 403 (53.8%) isolates to be *S. aureus*. Kirby-Bauer disc diffusion method was conducted to evaluate antibiotic resistance and 90.8% of *S. aureus* isolates were found to be resistant to at least three antibiotics, and therefore, classified as multidrug resistant. Of the antibiotics tested, 98% of the isolates demonstrated resistance towards penicillin G. High resistance was shown against different heavy metals, with 90% (196/217), 88% (192/217), 86% (188/217) and 84% (183/217) of the isolates resistant to 1500 µg/mL concentration of Cadmium (Cd), Zinc (Zn), Lead (Pb) and Copper (Cu) respectively. A total of 10 antimicrobial resistance and virulence genetic determinants were screened for all livestock associated *S. aureus* isolates. Methicillin-resistant *S. aureus* (MRSA) isolates were identified, by the presence of *mecC*, in 27% of the isolates with a significant relationship (*p* < 0.001)) with the host animal. This is the first report of *mecC* positive LA-MRSA in South Africa and the African continent. The gene for tetracycline resistance (*tetK)* was the most frequently detected of the screened genes with an overall prevalence of 35% and the highest prevalence percentage was observed for goats (56.76%) followed by avian species (chicken, duck and wild birds) (42.5%). Virulence-associated genes were observed across all animal host species. The study reports the presence of *luks/pv*, a gene encoding the PVL toxin previously described to be a marker for community acquired-MRSA, suggesting the crossing of species between human and livestock. The high prevalence of *S. aureus* from the livestock indicates a major food security and healthcare threat. This threat is further compounded by the virulence of the pathogen, which causes numerous clinical manifestations. The phenomenon of co-selection is observed in this study as isolates exhibited resistance to both antibiotics and heavy metals. Further, all the screened antibiotic and heavy metal resistance genes did not correspond with the phenotypic resistance.

## 1. Introduction

*Staphylococcus* (*S*.) *aureus* is one of the most clinically important pathogens of humans and animals, forming part of the normal commensal flora of humans and colonizing more than 30% of the population [1]. *S. aureus* is associated with numerous infections varying in severity, and has been reported to be life-threatening with mortality rates higher than those for AIDS, tuberculosis and viral hepatitis combined [2]. Recently, an eleven-month-old infant in Durban, KwaZulu-Natal was diagnosed with cellulitis caused by *S. aureus* with symptoms that included swelling in the forehead and eyes, increased body temperature, vomiting and fatigue (personal communication). In 2015, a food poisoning outbreak was reported in Italy where 57% of customers in a local restaurant presented with gastrointestinal symptoms including vomiting and diarrhoea, and upon further investigation, enterotoxigenic *S. aureus* was discovered in food handlers, kitchen surfaces and dessert [3]. The US Centre for disease control (CDC) has described *S. aureus* as an important cause of serious infections in humans due to community associated *S. aureus* (CA-SA) and this was further established when approximately 32% of samples obtained from children’s playground surfaces tested positive for *S. aureus* [4,5]. Healthcare associated infections have also been reported, to varying degrees, and recently *S. aureus* has been reported to be the most common causative pathogen in surgical site infections [6].

The versatility of *S. aureus* spreads to the colonization of companion animals, livestock as well as the food production system. An outbreak of mastitis caused by a strain of *S. aureus* was reported in a closed dairy herd [7]. Livestock are an important reservoir of *S. aureus* and pose a great zoonotic threat to the human population as it can be transferred to humans via contaminated animal products as well as occupational contact [8]. Alarmingly, numerous cases have been reported where humans were infected with livestock-associated *S. aureus* (LA-SA), such as a case wherein a six-month old baby was found to be infected with methicillin-resistant *S. aureus* (MRSA) [9]. The ability of LA-SA to infect human populations poses a huge burden on the healthcare system.

The virulence of *S. aureus* lies in the production of a repertoire of virulence factors, most commonly toxins which are substances produced by the organism and directly interfere with the host cells [10]. It is important to note that the role of a single virulence factor is difficult to establish and thus virulence is conferred by the interplay and synergistic effect of more than one factor. Numerous toxins that exacerbate virulence in *S. aureus* have been reported [10], with Panton-Valentine Leucocidin (PVL) being one of the most essential, potent and prevalent [10]. *LukS-PV*, a component of PVL, was detected in the lungs of patients infected with necrotizing pneumonia, postulating that PVL induces apoptosis and is directly involved in the pathology of necrotizing pneumonia [11]. Together with pathogenicity, a major cause for alarm is the drastic rising of antibiotic resistance of *S. aureus*. The World Health Organization (WHO) has described antibiotic resistance as one of the biggest challenges to global health and food security today [12]. Alarmingly, news headlines around the world have caused great panic highlighting cases from “Resistance to last resort antibiotic has now spread across the globe” (2016), to “Doctors warn serious threat to patients from almost untreatable superbug”(2017), to “Scots gran has all limbs cut off after paper cut leads to sepsis” (2018) and “Antibiotic resistance plan to fight urgent global threat” (2019) [13]. These headlines demonstrate the urgency to combat the challenge of antibiotic resistance.

*S. aureus* infections are most commonly treated using different antibiotics, including β-lactam antibiotics (such as penicillins, cephalosporins, and carbapenems) and non-β-lactam antibiotics (such as macrolides, azalides and fluoroquinolones) [14]. With the world experiencing a considerable increase in the consumption of antibiotics worldwide, with South Africa, Brazil and China accounting for 76% of the increase [15], MRSA has been coined the ‘superbug’.

According to the WHO, *S. aureus* is classified as a high priority superbug for which research and development of new antibiotics are required urgently [16]. Of major concern is that the world has seen a significant increase in the consumption of two last resort classes of antibiotics, carbapenems (45%) and polymyxins (13%) [15].

Apart from antibiotics, there are numerous other contaminants that threaten the health and security of livestock and humans. Heavy metals contaminate the environment from everyday processes such as the earth’s crust, paint runoff and the use of detergents. Numerous studies have reported heavy metal contamination in the environment, posing a threat to the produced foodstuff [17,18]. There are recommended heavy metal toxic limits for animals and humans [19] and the consumption of contaminated food poses major health threats to both humans and animals. Furthermore, the exposure of environmental bacteria to heavy metals allows for the emergence and selection of heavy metal resistant strains, which coupled with antibiotic resistance, makes treatment extremely complicated. Numerous studies have reported heavy metal resistant bacteria in contaminated environments and the reduced susceptibility of bacteria from humans and animals to heavy metals [20,21,22].

South African agriculture contributes a significant percentage to the Gross Domestic Product (GDP). Gross farming has witnessed a 10.2% increase estimated at R267 009 million as at June 2017 [23]. Further, animal products contributed approximately 47% to gross farming with the poultry and cattle industries contributing 15% and 13%, respectively [23]. This highlights the significance of livestock production and thus huge efforts are required to ensure that livestock production is optimal. The compounding of antibiotic resistance with heavy metal resistance poses a major challenge to both production and health, making infections very complicated and difficult-to-treat. The WHO has reported that the African region has the largest data gap on the prevalence of antimicrobial resistance and to the best of our knowledge, there is currently no study that evaluates antibiotic and heavy metal resistance in livestock in South Africa. Therefore, the current study aimed to investigate the prevalence of *S. aureus* in South African livestock and their environment using a one-health approach. Another objective of the study was to determine the virulence as well as the phenotypic and genetic characterization of antibiotic and heavy metal resistant isolates.

## 2. Materials and Methods

### 2.1. Ethical Approval

The study was approved by the Animal Research Ethics Committee of the University of Kwa-Zulu Natal (Reference numbers AREC/051/017M, AREC 071/017, AREC 014/018). The field sampling protocols, samples collected from animals, and the research were conducted in full compliance with Section 20 of the Animal Diseases Act of 1984 (Act No 35 of 1984) and were approved by the South African Department of Agriculture, Forestry and Fisheries DAFF (Section 20 approval reference number 12/11/1/5 granted to Prof Dr. ME El Zowalaty).

### 2.2. Sample Collection

**Figure 1:**
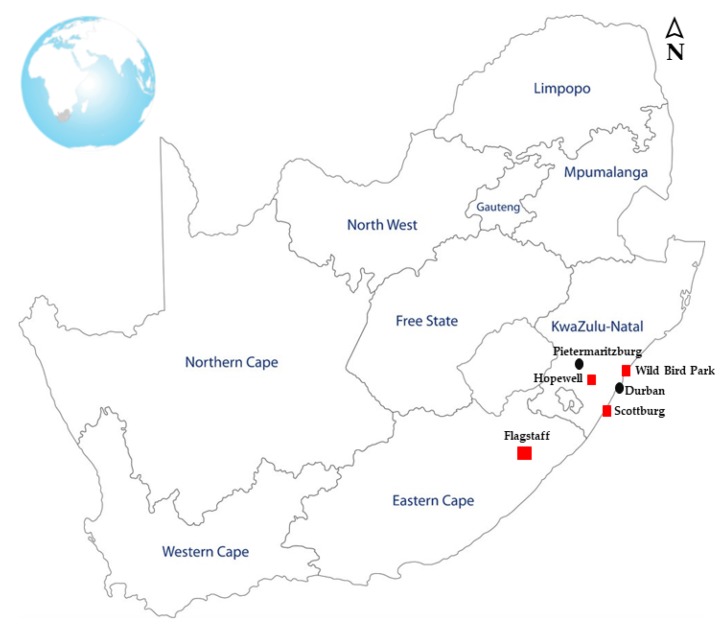
Geographic map of South Africa showing the sampling areas in this study. (Flagstaff: farm A and farm B); Scottburg; Hopewell and Wild Bird Park in Durban).

A total of 403 samples were randomly collected from 5 smallholder farms and a wild bird park in the Eastern Cape and KwaZulu-Natal Provinces in South Africa (Figure 1) from May 2018 to September 2018. The sampling period covered autumn, winter and spring seasons. The samples included avian (chickens, ducks and wild birds) (*n* = 142), pigs (*n* = 167), sheep (*n* = 28), horses (*n* = 5), goats (*n* = 51) and cows (*n* = 10). In a nutshell, sterile cotton swabs were used to collect oral, fecal, cloacal and environmental (water, animal feed, pens, crates, walls, floor) samples. These swabs were collected into plastic screw top tubes containing 10 mL of 0.1% (*w*/*v*) peptone water and tubes were stored on ice during transport to the laboratory at the Department of Genetics, Westville Campus of the University of KwaZulu-Natal for further analysis.

### 2.3. Sample Processing and Bacterial Isolation

The samples were inoculated in 0.1% (*w*/*v*) peptone water and incubated for 24 h at 37 °C. One hundred microliters of the culture was inoculated into 10 mL Brain Heart Infusion (BHI) broth and subsequently incubated for 24 h at 37 °C. Further, a loopful of sample was streaked onto *S. aureus* ChromoSelect agar base (Sigma-Aldrich, Cat No. 05662, Bangalore, India,) supplemented with 50 mL/L egg yolk tellurite emulsion (Sigma-Aldrich, Cat No. 75208, Bangalore, India). Presumptive *S. aureus* pure colonies (characterized by a blackish colour) were selected and subsequently inoculated into BHI and incubated for a further 24 h at 37 °C for further enrichment. Sixty percent glycerol stocks of the pure colonies were prepared and cultures were stored at −80°C while working stocks were stored at −20 °C and used for molecular and phenotypic characterization tests.

### 2.4. DNA Extraction and Molecular Identification of S. aureus

DNA from all presumptive *S. aureus* isolates was extracted using the conventional boiling method as previously described [24] and DNA was subsequently quantified using Nanodrop spectrophotometer. All DNA extracts were stored at −20 °C until used for molecular confirmation of virulence, antimicrobial and heavy metal resistance genes. Polymerase chain reaction (PCR) was carried out to confirm *S. aureus* isolates by amplifying the *nuc* gene (amplicon 270 bp) and *S. aureus* ATCC 29213 was used as a positive control. Briefly, PCR was carried using a total volume of 15 µL containing 7 µL DreamTaq Green Master Mix (Thermo Fisher Scientific, Cat: K1081, Thermo Fisher Scientific Baltics UAB, Vilnius, Lithuania); 0.5 µL *nuc* forward primer; 0.5 µL *nuc* reverse primer; 4 µL template DNA and 3 µL dH_2_O. *nuc* gene primers used are detailed in Table 1. Amplification was carried out using a thermocycler (BioRad, California, USA) according to the following PCR steps of an initial denaturation for 3 min at 95 °C followed by 36 cycles consisting of denaturation for 30 s at 95 °C, annealing for 45 s at 68 °C, extension for 1 min at 72 °C and final extension for 5 min at 72 °C. Finally, PCR products were visualized on 2% agarose gel, stained with ethidium bromide, using electrophoresis for 1 h at 70 volts with 1X TAE used a medium buffer and visualized under UV light using the Bio ChemiDoc imaging system (BioRad, California, USA).

### 2.5. Antimicrobial and Heavy Metal Susceptibility Testing

Antimicrobial susceptibility testing of the confirmed *S. aureus* isolates was performed using the Kirby-Bauer disk diffusion method in accordance with the CLSI as previously described [34]. The following antibiotics were used: penicillin G (P; 10 IU), chloramphenicol (C; 30 µg), cefoxitin (FOX; 30 µg), gentamicin (CN; 10 µg), tetracycline (TE; 30 µg), trimethoprim/sulfamethoxazole (SXT; 25 µg), ciprofloxacin (CIP; 5 µg); erythromycin (E; 15 µg), and rifampicin (RIF; 5 µg). Multidrug resistance (MDR) was determined when an isolate was resistant to two or more antibiotics [30,35,36]. Briefly, a loopful of the glycerol stock sample was inoculated into BHI and incubated at 37 °C for 24 h. One hundred microliters of the sample were streaked onto Mueller-Hinton agar plates using a sterile cotton swab. The plates were allowed to dry, and the antibiotic discs were applied onto the inoculated agar plates which were subsequently incubated at 37 °C for 24 h. The zones of inhibition were measured and results were interpreted as sensitive (S), intermediate (I) or resistant (R) according to the CLSI guidelines [34].

Heavy metal susceptibility testing was performed following a protocol previously described [37]. Briefly, each isolate was evaluated on Mueller-Hinton agar plates supplemented with increasing concentrations of each heavy metal salt. The starting concentration of the heavy metal was 50 µg/mL and the concentration was increased by 50 µg/mL intervals until the isolate failed to grow. Further, higher concentration plates were inoculated with isolate from the previous concentration [21] and plates were incubated at 37 °C for 24 h. The following heavy metal salts were used in this study to test for heavy metal tolerance: Copper sulphate pentahydrate (CuSO_4_·5H_2_O) (Merck, Darmstadt, Germany), Cadmium nitrate tetrahydrate (Cd(NO_3_)_2_·4H_2_O) (Sisco Research Laboratories, Maharashtra, India); Zinc sulfate (ZnSO_4_·7H_2_O) (Merck Life Science, Mumbai, India) and Lead nitrate (PbNO_3_)_2_ (Merck Life Science, Mumbai, India). 

### 2.6. Molecular Detection of Virulence, Antimicrobial and Heavy Metal Resistance Genes

The isolates that tested positive for the *nuc* gene were further screened for virulence, antimicrobial and heavy metal resistance genes. Briefly, a multiplex PCR was conducted in a total volume of 15 µL consisting of containing 7 µL DreamTaq Green Master Mix (Thermo Fisher Scientific, Cat: K1081, Thermo Fisher Scientific Baltics UAB, Vilnius, Lithuania); 0.5 µL primer (forward); 0.5µL primer (reverse); 4 µL template DNA and 3 µL dH_2_O. Primers used are shown in Table 1. Amplification was carried out using a thermocycler (BioRad, California, USA) according to the following PCR steps of an initial denaturation for 3 min at 95 °C followed by 36 cycles consisting of denaturation for 30 s at 95 °C, annealing for 45 s at various temperatures (Table 1), extension for 1 min at 72 °C and final extension for 5 min at 72 °C. PCR conditions were slightly modified following temperature optimizations. PCR products were visualized on a 2%, agarose gel, stained with ethidium bromide using 1X TAE as a buffer medium. The ChemiDoc MP imaging system (BioRad, California, USA) was used to visualize the gels under UV light. 

### 2.7. Statistical Analysis

Relationships between data observed throughout this study were measured using the IBM SPSS, version 25 statistical package. Further, relationships between the discrete variables were determined by the Chi-square test with *p* < 0.05 considered as statistically significant. The effects of location where the samples were collected from an animal host in the presence of *S. aureus*. were investigated using the Fischer’s exact test. The Fischer’s exact test is a parametric test of significance that is used in the place of a Chi- Square test in 2 by 2 tables. Pearson’s correlation test was implemented to evaluate the strength and direction of the relationship between the antibiotic resistance, virulence and heavy metal resistance genes.

## 3. Results

### 3.1. Prevalence of S. aureus

In the present study, a total of 217 (53.8%) out of 403 samples were confirmed to be *S. aureus* by *nuc* gene PCR methods. Table 2 shows the number of samples collected per host animal and the prevalence of *S. aureus* in each animal host species. Table 3 shows the number of *S. aureu*s positive isolates that were isolated from samples collected from different wild bird species. A 2-tailed Pearson’s correlation test was conducted to determine the strength and association between microbial and molecular methods for the detection of *S. aureus*. Results showed a significant (*p* < 0.001), positive correlation (52.8%).

### 3.2. Antimicrobial and Heavy Metal Resistance Susceptibility Testing

All the 217 *S. aureus* isolates were tested against nine antibiotics. The antimicrobial resistance profiles of the isolates to antimicrobial agents are shown in Table 4. Ciprofloxacin showed the lowest resistance rate of 14%. In the present study, the majority of isolates were classified as MDR-SA, where 216 (99.5%) isolates were resistant to two or more antibiotics, while 197 (90.8%) isolates were resistant to three or more drugs and were defined as multidrug resistant, and two (0.9%) isolates were resistant to all nine antibiotics.

**Figure 2:**
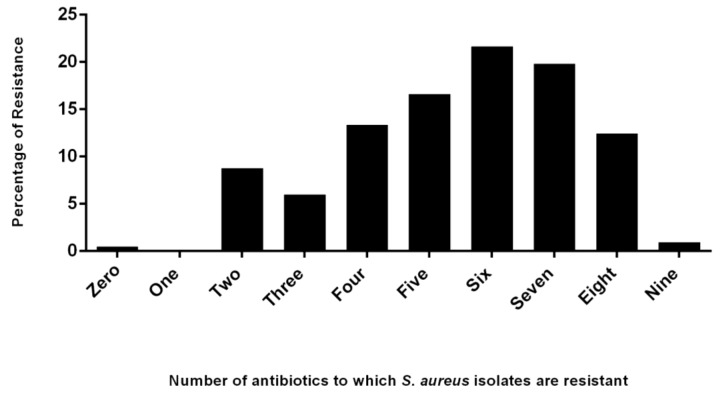
Resistance of *S. aureus* isolates to one of more antibiotics in the present study

The resistance of *S. aureus* isolates to one or more antibiotics is shown in Figure 2. It was found that 2 (0.9%) isolates were resistant to all tested nine antibiotics and one (0.5%) isolate was susceptible to all tested nine antibiotics. It was found that 19 (8.8%), 13 (5.9%), 29 (13.4), 36 (16.6%), 47 (21.7%), 43 (19.8%), and 27 (12.4%) isolates were found to be resistant to two, three, four, five, six, seven and eight antibiotics respectively (Figure 2). In the present study, 98.6% and 90.8% of *S. aureus* isolates were found to be resistant to at least two and three antibiotics respectively, and were therefore classified as multidrug resistant. 

Further, all the 217 *S. aureus* isolates were tested for heavy metal susceptibility against four heavy metals with increasing concentrations up to a concentration of 1500 µg/mL. High resistance rates were found against the different heavy metals with 88.9% (193/217), 84% (182/217), 86.2% (187/217), and 88.4% (192/217) of the samples were resistant to 1500 µg/mL concentration of Cd, Cu, Pb, and Zn respectively (Table 5).

### 3.3. Virulence, Antimicrobial and Heavy Metal Resistance Genes

**Figure 3:**
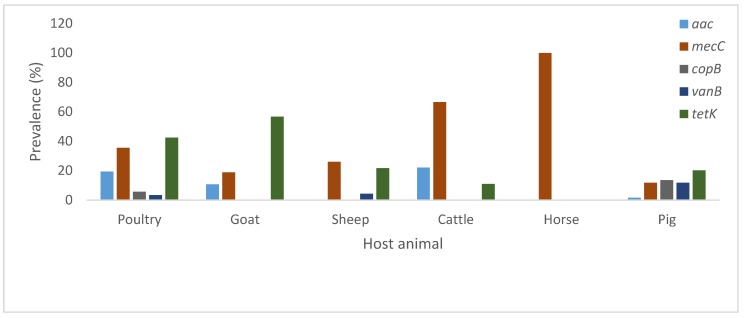
Antimicrobial resistance determinants of *S. aureus* isolated from livestock in South Africa.

**Figure 4:**
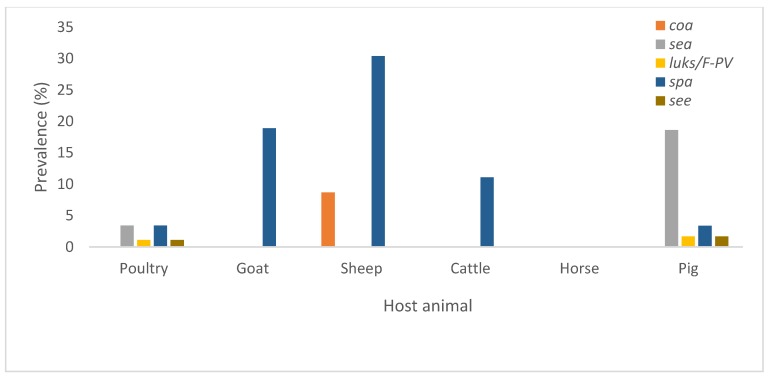
Virulence associated genes detected in *S. aureus* isolated from livestock in South Africa.

Samples were screened for a total of 10 genetic determinants associated with virulence as well as antimicrobial and heavy metal resistance. Figure 3 highlights the prevalence rates of antimicrobial resistance genes in the tested *S. aureus* isolates. The most prevalent gene was *tetK* (35.3%) conferring resistance to tetracycline. The second highest prevalent gene was *mecC* (27%)*,* classifying these isolates as MRSA. The most prominent genetic profile for virulence was *sea-luks/pv-spa*, with the antimicrobial resistance profile being *aac-mecC-tetK.* Figure 4 shows the prevalence of different virulence genes in *S. aureus* isolated from each animal host species and Table 6 highlights the overall number of screened genes per livestock animal species. Statistical analysis highlighted associations between the different variables with genetic determinants (Table 7) and relationships between the genetic determinants (Table 8).

## 4. Discussion

*S. aureus* is an important pathogen that can cause a myriad of infections. It has been reported to have a zoonotic potential and causes significant clinical problems [9], causing major alarms in both the food security and healthcare sectors. The current study reports an overall 53.8% prevalence of *S. aureus* in South African livestock production systems. The significant (*p* < 0.05)) and positive correlation (52.8%) between the conventional and molecular methods of *S. aureus* detection highlight the importance of using both methods in parallel during microbial identification and diagnosis. Although molecular detection methods are less laborious, more sensitive and reliable, it may be cost-effective to start identification using conventional microbiological methods. The prevalence results in the current study are in concordance with a local study where *S. aureus* was isolated from approximately 54% of broiler chicken samples [30]. However, this prevalence raises alarms as it is much higher than other global reports of a 15% *S. aureus* prevalence from smallholder dairy farms in Tanzania [38], a 37.2% prevalence in Chinese aquatic products [39] and 40.94% and 36.23% in Korean imported and domestic meat products, respectively [40]. The presence of *S. aureus* in livestock poses a great challenge to food security, because most of the farms where samples were collected in this study are smallholder farms that supply food of animal origin to the underprivileged rural communities. Fecal material is regarded as a vehicle for pathogen transmission and spread [30], and thus a relatively high prevalence poses a threat to human population.

The injudicious use of antibiotics has caused a major global burden on the healthcare system. Global antibiotic consumption has shown an increase of 65% from 2000 to 2015 in low and middle income countries [41]. The WHO has reported the list of infections that are now difficult to treat is growing at an alarming rate due to antibiotics becoming less effective, making these infections sometimes untreatable [12]. Further, in a previous communication, it was reported that antibiotics (such as bacitracin, tetracyclines and quinolones) are misused on a no-prescription basis for livestock and backyard poultry farming in rural areas in South Africa [42]. In addition, livestock and piggery farmers use tylosin (a macrolide antibiotic) and depomycin (a beta-lactam antibiotic) for non-therapeutic purposes (Personal communication). The unabated frequent exposure to antibiotics has created a suitable selective environment for the emergence of antibiotic resistant bacteria. This is evident from the results of this study where 98% of the *S. aureus* isolates were resistant to penicillin G and the lowest resistance rate was 77% towards rifampicin. The resistance towards rifampicin is quite concerning because previously this antibiotic has been used extensively in South Africa for the treatment of tuberculosis until there was an evolution of multidrug resistant tuberculosis about a decade ago in South Africa [43]. The complexity of antimicrobial resistance requires the implementation of one-health approaches to determine the interplay between bacterial pathogens at animal–human-environment interfaces, which will help us to control the escalating antimicrobial resistance. The current study showed 80% and 77% resistance towards tetracycline and macrolides, respectively, which was higher than previously reported rates of 39% and 23%, respectively [44] Recently, similar results were reported with resistance rates of 94.6% and 10–15% for penicillin G and gentamicin, respectively [44]. However, higher resistance rate (56.7%) was observed for ciprofloxacin [44] as compared to the resistance rate of 14% in the current study. The current findings highlight the rapidly increasing resistance to commonly used antibiotics and further raises concerns as the consumption of last-resort antibiotics such as carbapenems and polymyxins has increased to 45% and 13%, respectively [15].

Alarmingly, the prevalence rate of multidrug resistance in the present study is high and the majority of isolates were defined as multidrug resistant. This reflects the high prevalence of such resistance in the farms where the isolates were collected. The detection of MDR Livestock-associated *S. aureus* strains poses high risk to humans who are in contact with farm animals and may result in severe infections [45,46]. Several studies worldwide have reported the detection of LA-SA including MDR and MRSA strains among swine [47,48,49], poultry [50,51], bovine [52,53,54], equine [55], sheep [56,57,58], goat [59,60,61], and animal workers [50,52,62,63]. In the present study, 59 (27%) of the 217 *S. aureus* isolates harboured the *mecC* gene and were identified as MRSA. Different studies reported the detection of MRSA in livestock from different parts of the world. In the present study, 14.3% of MRSA isolates were identified in avian species including chicken, duck and wild birds, while 3.2% of the MRSA isolates were identified in swine, 2.7% in cow and sheep, 3.2% in goat, and 0.9% in horses (Table 6).

Similar to antibiotics, exposure of bacteria to heavy metals results in tolerance, as was evident in this study. The current findings are in concordance with previous studies where high tolerance rates towards heavy metals by *S. aureus* were reported [37]. Further, other studies have reported that samples from poultry (chicken and turkey) meat products were contaminated with heavy metals at levels that are above the permissible limits, these included Cd (0.0–5.68 mg/kg), Ni (0.13–7.93 mg/kg), Cr (0.01–3.43 mg/kg) and Pb (0.01–4.60 mg/kg) [18]. This highlights the threats posed by heavy metals as they can cause major toxicity to humans upon consumption of the contaminated food. The development of antimicrobial and heavy metal resistant bacteria, specifically *S. aureus*, further complicates treatment and poses major threats to human health and in medical practice. The co-selection of antimicrobial and heavy metal resistance is supported by a study showing the co-existence of antibiotic and heavy metal resistance-related genes in composite staphylococcal cassette chromosome (SCC) [64]. Further, it was suggested that, because a composite SCC island can be freely transferred between hosts and other staphylococcal species, farming and food producing environments including soil, water and feed should be carefully monitored as resistance to both antibiotics and heavy metals can be easily disseminated [64]. Moreover, an insightful review on the relevance of foodborne pathogens to the co-selection of resistance to antibiotic, biocides and heavy metals was reported [65].

Environmental heavy metal contamination can be attributed to a myriad of processes, one of which is runoff from detergents. A study conducted in Irish domestic wastewater showed laundry detergents as a source of heavy metals [66]. The study showed that in Irish municipal wastewater, the heavy metal contribution from detergents were 31.9%, 0.24% and 0.30% for Cd, Cu and Zn, respectively. Similarly, concentrations of Cd, Cr, Ni and Pb were 3.42 mg/kg, 3.13 mg/kg, 4.67 mg/kg and 1.35 mg/kg, respectively were observed in detergents used [67]. Detergents are used in everyday application including in farms and food production environments for a multitude of purposes as well as for personnel use. Therefore, the development of heavy metal resistance by bacteria in the environment is inevitable. Subsequently, the current study further enforces the co-selection phenomenon as *S. aureus* isolates from livestock and their environments showed high phenotypic resistance to both antibiotics and heavy metals. The compounding of resistance into one ‘superbug’ further highlights the major problems facing the food security and healthcare sectors as infections become more complicated and very difficult-to-treat effectively, and may results in emerging untreatable staphylococcal infections.

Molecular characterization plays a fundamental role in the understanding and timely detection of different microorganisms. As such, numerous studies have been undertaken to elucidate the genetics of *S. aureus* including genes that confer resistance to antibiotics, heavy metals as well as virulence genes. MRSA strains are major health threat and were isolated from infected patients, livestock, aquatic products, different environmental sources including wastewater [39,68,69]. The predominant target gene to be screened to detect methicillin resistance is *mecA,* however, numerous studies have reported *S. aureus* isolates that exhibited phenotypic resistance to methicillin but were found to be negative for the *mecA* gene [70,71,72]. Similarly, in the current study, *mecA* was not detected in the tested isolates and therefore, the present study represents the first report on the detection of *mecC*-positive LA-MRSA in South Africa and the African continent at the time of this report. Previously, no study reported the detection of *mecC* in *S. aureus* or *Staphylococci* isolates in Africa [73,74,75,76,77,78,79,80,81,82]. The finding therefore confirms that livestock animals might act as a *mecC*-MRSA reservoir. This is congruent with previous studies that have reported *mecC* to be associated more frequently with LA-MRSA suggesting a zoonotic reservoir [73,83,84,85,86]. The detection of *mecC*-MRSA in the present study highlights the diagnostic importance of screening for *mecC* in *mecA*-negative MRSA, which was previously recommended [56,76].

Interestingly, the association tested for by the Fischer’s exact test (Table 7) demonstrated a significant association (*p* < 0.05) between *mecC* and the host species but not with the other variables such as location (*p* > 0.05) and sample material (*p* > 0.05), further suggesting that livestock are a significant host of *mecC-*positive MRSA compared to humans. A study from Spain confirmed the presence of *mecC-*positive MRSA in a patient that died from sepsis [87], indicating the ability of LA-MRSA to cause detrimental effects in humans which is a major cause for alarm. Further, in the present study, the phenotypic resistance observed towards cefoxitin was significantly higher than the prevalence of genotypic basis of resistance tested. Several mechanisms have been reported which contributed to the development of methicillin resistance such as the PBP2a regulated by the *blaZ-blaI-blaR1* and *mecA-mecI-mecRI* systems [88,89], while the expression of the *femA* gene has been shown to be essential in the expression of methicillin resistance [90]. This lends an explanation to the difference in the phenotypic and genotypic results in the current study. The high phenotypic MRSA results may be due to one or more genetic mechanisms that were not explored in this study. It was recently reported that misuse of antibiotics belonging to the tetracycline and quinolones classes on a non-prescription basis for backyard poultry and livestock is frequent in several rural areas in South Africa [42]. This has resulted in high levels of resistance to tetracycline to be expected due to the over exposure, it is therefore not surprising in the present study that *tetK* gene showed the highest prevalence (35%) among all the screened genes, showing significant (*p* < 0.05) associations with the host species, sample material and location. It was previously reported that tetracycline resistance is one of the most frequent resistances in *S. aureus* from poultry farming [91]. This is further supported by the 37% prevalence of *tetK* in abattoir broiler chickens [30]. The reported prevalence in the current study suggests the urgent need for more stringent protocols in the use of tetracycline. *S. aureus* of avian origin in the present study showed the highest prevalence rate of *tetK* gene where 48.68% (37/76) of the isolates that were positive for the gene were from avian species. These results are expected as tetracycline is injudiciously used in South African poultry farming. It was previously reported that genes that confer heavy metal resistance are frequently present in LA-MRSA, this was supported by a 24.3% prevalence of *copB* among LA-MRSA isolates in Europe [92]. Plasmids that confer multidrug resistance by harbouring heavy metal genes, such as *copB*, as well as antibiotic resistance genes have been reported, suggesting co-selection and dissemination of genes that promote life-threatening *S. aureus* infections [93]. As such, presently, a 5% prevalence of *copB* is reported with the gene significantly associated (*p* < 0.05) with both sample material and location (Table 7). This further accentuates that resistance to heavy metals is developed through environmental contaminants. Moreover, of interest is that *copB* has a positive and significant relationship with *vanB,* a gene that showed a 0.9% prevalence and confers resistance to vancomycin (Table 8). The significant relationship between the two genes further accentuates the co-selection of heavy metal and antibiotic resistance genes. Further, this study reports an 11% frequency of aminoglycoside (*aac(6*′*)-aph(2*″*).* Although a previous study reported a 43% *aac(6*′*)-aph(2*′*)* prevalence [27], the low frequency in the current study is still cause for alarm as the co-existence with other antimicrobial resistance genes requires large scale and frequent surveillance.

The prevalence of numerous virulence associated genes in *S. aureus* has been frequently investigated globally [30,39,40,94]. A prevalence rate of 27% for the *sea* gene was observed from clinical isolates in patients in Iran [95]. Interestingly, German porcine did not show any presence of the enterotoxin genes [49]. Similarly, the current study showed a relatively low prevalence rate of 6.4% for *sea* and 6% for *see* genes. Moreover, statistical analysis shows a significant relationship (*p* < 0.05) between the two genes. The low frequency of these genes suggest the low capacity of these isolates to induce toxin-mediated disease [49]. This is unsurprising because the host species sampled were asymptomatic, however, with the high mutation as well as cross-species infection ability of *S. aureus*, vigilant protocols are required for the monitoring of colonization to prevent infection that could be detrimental to both human and animals.

The virulence factor, PVL is encoded for by numerous genes and has been reported to be a stable marker for CA-MRSA [96,97]. A prevalence rate of 11.1% was described in clinical isolates from patients with the gene associated with increased disease severity of *mecA-*positive *S. aureus* strains [97]. The association of PVL genes with clinical MRSA versus LA-MRSA was further emphasized where 20% of human isolates were PVL- positive while none of the livestock isolates had the gene [98]. However, a recent study has described a relatively low occurrence of PVL gene in *S. aureus* isolated from livestock [99]. Similarly, the current study shows a prevalence rate of 0.9% for *luks/pv* with a non-significant relationship with *mecC.* This finding highlights the potential occupational risk of transfer of virulent *S. aureus* isolates between humans and livestock in the farm settings. Host-switching between human and livestock clones has been reported, resulting in the evolution of *S. aureus* and the emergence of clones with complicated virulence and antimicrobial resistance [100].

Among the virulence factors screened, *spa* and *coa* showed prevalence rates of 9.17% and 0.9%, respectively. These results are congruent with a study that reported prevalence rates of 11% and 5% from *S. aureus* isolated from chickens in abattoirs [30]. The virulence gene *coa* codes for the coagulase, and the low frequency of this gene indicates that most of the isolates in the current study are coagulase-negative. Pearson’s correlation reports positive and significant relationships between *coa* and *spa* (*p* = 0.000) (Table 8), supporting the coexistence of virulence factors, and thus explains the low frequencies of all the virulence genes.

The findings of the current study showed lower frequencies of the screened genetic determinants as compared to the observed phenotypic characteristics. This can be attributed to the numerous genes that can contribute to a certain phenotype. Based on the current study, investigations of the *mecA* gene yielded negative results, however, *mecC* showed a relatively high prevalence. As such, the genes that may have contributed to the observed phenotypes in this study, most significantly antibiotic resistance, may not have been part of this study. Moreover, the low frequencies observed for the virulence genes do not necessarily mean there is no cause for action. The implementation of strict surveillance systems and biosecurity practices in livestock production systems utilizing one-health approaches are significantly and increasingly required to limit and contain the spread of LA-SA as well as to prevent any possible detrimental outbreaks.

## 5. Conclusions

Virulent and multidrug resistant *S. aureus* including MRSA isolates were detected in South African livestock production systems as well as their environments. These findings highlight the importance of surveillance for LA-SA and MRSA in food chain animals. The antibiotic resistance rates observed highlight the importance of implementation of strict policies and strategies on the prudent use of antibiotics by the public as well as the farming sector. The government, physicians and farming industries are urged to limit imprudent antibiotic use in order to save the existing potential antibiotics, especially in developing countries. The detection of LA-MRSA further accentuates the potential risks for occupational exposure and life-threatening infections, with treatment further complicated by the complexity of antibiotic and heavy metal resistance. Virulence associated genes play an important role in the development of disease, the presence of the PVL toxin, a previously CA-MRSA marker, highlights the potential of cross transmission of *S. aureus* species between humans and animals. However, *S. aureus* in the current study were screened for two enterotoxins, thus further in-depth genetic analysis is required including whole genome sequencing to further elucidate on the genetic components of these isolates. To the best of our knowledge, this is the first study in South Africa to determine antibiotic and heavy metal resistance in LA-SA. In addition, the present study is the first to report the detection of *mecC-*positive MRSA in Africa. The findings of the current study will significantly contribute to the existing body of knowledge of research towards public and veterinary health as well as food safety and security, especially in developing countries and highlight the importance of implementing one-health approaches to reduce the ongoing spread of antimicrobial resistance.

## Figures and Tables

**Table 1:** Primer sequences used to amplify specific virulence, antibiotic and heavy metal resistance genes in this study.

Target	Gene	Primer Sequence	Tm (°C)	Amplicon Size (bp)	Reference
**Thermonuclease**	*nuc*	**F**-GCGATTGATGGTGATACGGTT **R**-AGCCAAGCCTTGACGAACTAAAGC	68	270	[25]
**Methicillin**	*mecC*	**F**- GAAAAAAAGGCTTAGAACGCCTC **R**- GAAGATCTTTTCCGTTTTCAGC	54	138	[26]
**Aminoglycosides**	*aac(6′)-aph(2″)*	**F**-TAATCCAAGAGCAATAAGGGC **R**-GCCACACTATCATAACCACTA	61	227	[27]
**Tetracycline**	*tetK*	**F**-TCGATAGGAACAGCAGTA **R**-CAGCAGATCCTACTCCTT	57	169	[28]
**Vancomycin**	*vanB*	**F**- GTGACAAACCGGAGCGAGGA **R**- CCGCCATCCTCCTGCAAAAAA	46	433	[29]
**Leukocidin**	*LukS/F-PV*	**F**-ATCATTAGGTAAAATGTCTGGACATGATCCA **R**-GCATCAAGTGTATTGGATAGCAAAAGC	56	443	[30]
**Others**	*spa*	**F**-CAAGCACCAAAAGAGGAA **R**-CACCAGGTTTAACGACAT	57	180	
	*coa*	**F**-CGAGACCAAGATTCAACAAG **R**-AAAGAAAACCACTCACATCA	61	730	
**Enterotoxins**	*sea*	**F**- CCTTTGGAAACGGTTAAAACG **R**-TCTGAACCTTCCCATCAAAAAC	56	127	[31]
	*see*	**F**-TAGATAAAGTTAAAAAACAAGC **R**-TAACTTACCGTGGACCCTTC	46	170	[32]
**Copper**	*copB*	**F**-TAGTGGCCATGCACATCATC **R**-CCACCAGACAAGAACGGTTT	60	201	[33]

**Table 2:** Prevalence of *Staphylococcus aureus* in samples collected from livestock animals in South Africa between May–September 2018.

Animal Host	Flagstaff				Scottburg						Hopewell						Total
	Oral	Fecal	Feed	Soil	Water	Oral	Fecal	Feed	Soil	Water	Oral	Fecal	Feed	Soil	Water	Other *	
**Chicken**	0	0	0	0	0	31/40	29/40	-	3/5	3/5	0	0	0	0	0	0	66/90
**Ducks**	0	4/10	0	0	0	0	0	0	0	0	0	0	0	0	0	0	4/10
**Cow**	0	4/5	0	5/5	0	0	0	0	0	0	0	0	0	0	0	0	9/10
**Goats**	0/1	7/9	0	5/6	2/6	13/15	10/14	0	0	0	0	0	0	0	0	0	37/51
**Sheep**	10/12	7/10	0	6/6	0	0	0	0	0	0	0	0	0	0	0	0	23/28
**Horses**	0	2/5	0	0	0	0	0	0	0	0	0	0	0	0	0	0	2/5
**Pigs**	13/17	9/17	4/9	9/9	5/6	0	0	0	0	0	2/10	7/35	4/18	0	0	6/46	59/167
**Total**	23/30	33/56	4/9	25/26	7/12	44/55	39/54	0	3/5	3/5	2/10	7/35	4/18	0	0	6/46	200/361

* Other: samples collected from the animals’ environment such as pens, crates, floor, railing, equipment, and roof.

**Table 3:** Prevalence of *S. aureus* in wild bird samples collected from a bird park in Durban, South Africa in May 2018.

Wild Bird Species	Durban			
	Oral	*S. aureus*	Fecal	*S. aureus*
**Scarlet ibis**	8	4	8	4
**African Spoonbill**	3	2	3	0
**Fulvis Whistling duck**	2	0	2	0
**Carolina duck**	2	1	2	2
**Bahama pintail**	2	1	2	0
**Fireback pheasant**	1	0	1	1
**Whiteface whistling duck**	1	1	1	0
**Mandrin duck**	1	1	1	0
**Yellow bale duck**	1	0	1	0
**Total**	21	10	21	7

**Table 4:** Antimicrobial susceptibility test results for all *S. aureus* isolates evaluated in this study.

Antibiotic Class	Antibiotic	Resistance Phenotypes		
		Resistant (R)	Intermediate (I)	Susceptible (S)
**β-lactam**	Penicillin G (10 IU)	98.1	0	1.9
	Cefoxitin (30 µg)	94.5	0	5.5
**Aminoglycoside**	Gentamicin (10 µg)	19	21	60
**Quinolone**	Ciprofloxacin (5 µg)	14.3	16.6	69.1
**Macrolides**	Erythromycin (15 µg)	76.9	5.1	18.0
**Tetracycline**	Tetracycline (30 µg)	79.6	1.4	19
**Phenicols**	Chloramphenicol (30 µg)	30.7	15.3	54
**Sulfonamides**	Trimethoprim-sulfamethoxazole (25 µg)	60.9	4.7	34.4
**Other**	Rifampicin (5 µg)	76.9	3.8	19.4

**Table 5:** Prevalence of isolates growing at increasing heavy metal concentrations.

Heavy Metal	No. of Samples with Growth at Each Concentration				
	50 µg/mL	100 µg/mL	500 µg/mL	1000 µg/mL	1500 µg/mL
**Cadmium (Cd)**	200 (92.2%)	198 (91.2%)	193 (88.9%)	193 (88.9%)	193 (88.9%)
**Copper (Cu)**	217 (100%)	217 (100%)	182 (84%)	182 (84%)	182 (84%)
**Lead (Pb)**	217 (100%)	217 (100%)	187 (86.2%)	187 (86.2%)	187 (86.2%)
**Zinc (Zn)**	196 (90%)	196 (90%)	194 (89.4%)	193 (88.9%)	192 (88.4%)

**Table 6:** Number of antimicrobial resistance and virulence associated genes detected in South African livestock samples.

Host	Genetic Determinant									
	*see*	*coa*	*sea*	*luks/F-PV*	*spa*	*mecC*	*copB*	*aac*	*vanB*	*tetK*
**Avian**	1	0	3	1	3	31	5	17	3	37
**Goat**	0	0	0	0	7	7	0	4	0	21
**Sheep**	0	2	0	0	7	6	0	0	1	5
**Cattle**	0	0	0	0	1	6	0	2	0	1
**Horse**	0	0	0	0	0	2	0	0	0	0
**Pig**	1	0	11	1	2	7	8	1	7	12
**Total**	2	2	14	2	20	59	13	24	11	76

**Table 7:** Fischer’s exact test *p*-values to show the association between variables with genetic determinants screened.

Variables	Genes									
	*aac*	*coa*	*sea*	*Luks/PV*	*spa*	*mecC*	*see*	*vanB*	*copB*	*tetK*
**Host Species**	0.001 *	0.057 **	0.07 **	1.000 **	0.001 *	0.000 *	0.005 *	0.290 **	0.083 **	0.000 *
**Sample material**	0.472 **	0.554 **	0.05 *	0.189 **	0.200 **	0.361 **	0.042 *	1.000 **	0.045 *	0.004 *
**Location**	0.000 *	0.651 **	0.035 *	1.000 **	0.627 **	0.075 **	0.005 *	0.45 **	0.048 *	0.00 *

* significant at the 0.05 level (2-tailed); ** not significant at the 0.05 level (2-tailed).

**Table 8:** Pearson’s correlation and *p*-values computed to determine the relationship between the different genetic determinants.

	*aac*	*coa*	*sea*	*Luks/pv*	*spa*	*mecC*	*see*	*vanB*	*copB*	*tetK*
***aac***	1	−0.034 (0.618)	−0.093 (0.174)	−0.034 (0.618)	0.040 (0.558)	0.082 (0.231)	−0.089 (0.191)	−0.034 (0.618)	−0.081 (0.232)	0.076 (0.263)
***coa***	−0.034 (0.618)	1	−0.025 (0.711)	−0.009 (0.892)	0.303 ** (0.000)	0.158 * (0.020)	−0.024 (0.721)	−0.009 (0.892)	−0.022 (0.744)	−0.072 (0.294)
***sea***	−0.093 (0.174)	−0.025 (0.711)	1	0.171 * (0.012)	−0.019 (0.783)	0.008 (0.905)	0.329 ** (0.000)	−0.025 (0.711)	0.110 (0.105)	−0.038 (0.578)
***Luks/pv***	−0.034 (0.618)	−0.009 (0.892)	0.171 * (0.012)	1	0.136 * (0.045)	−0.059 (0.388)	−0.024 (0.721)	−0.009 (0.892)	−0.022 (0.744)	−0.072 (0.294)
***spa***	0.040 (0.558)	0.303 ** (0.000)	−0.019 (0.783)	0.136 * (0.045)	1	0.020 (0.768)	−0.080 (0.238)	−0.031 (0.653)	−0.001 (0.988)	0.030 (0.660)
***mecC***	0.082 (0.231)	0.158 * (0.020)	0.008 (0.905)	−0.059 (0.388)	0.020 (0.768)	1	0.064 (0.348)	−0.059 (0.388)	−0.047 (0.493)	−0.064 (0.352)
***see***	−0.089 (0.191)	−0.024 (0.721)	0.329 ** (0.000)	−0.024 (0.721)	−0.080 (0.238)	0.064 (0.348)	1	0.382 ** (0.000)	0.207 ** (0.002)	−0.025 (0.716)
***vanB***	−0.034 (0.618)	−0.009 (0.892)	−0.025 (0.711)	−0.009 (0.892)	−0.031 (0.653)	−0.059 (0.388)	0.382 ** (0.000)	1	0.198 ** (0.003)	0.029 (0.668)
***copB***	−0.081 (0.232)	−0.022 (0.744)	0.110 (0.105)	−0.022 (0.744)	−0.001 (0.988)	−0.047 (0.493)	0.207 ** (0.002)	0.198 ** (0.003)	1	−0.127 (0.061)
***tetK***	0.076 (0.263)	−0.072 (0.294)	−0.038 (0.578)	−0.072 (0.294)	0.030 (0.660)	−0.064 (0.352)	−0.025 (0.716)	0.029 (0.668)	−0.127 (0.061)	1

* Correlation is significant at the 0.05 level (2-tailed); ** Correlation is significant at the 0.01 level (2-tailed).
